# Feasibility of a randomized single-blind crossover trial to assess the effects of the second-generation slow-release dopamine agonists pramipexole and ropinirole on cued recall memory in idiopathic mild or moderate Parkinson’s disease without cognitive impairment

**DOI:** 10.1186/s40814-017-0154-7

**Published:** 2017-07-06

**Authors:** Thomas A. Shepherd, Nicola M. J. Edelstyn, Laura Longshaw, Julius Sim, Keira Watts, Andrew R. Mayes, Michael Murray, Simon J. Ellis

**Affiliations:** 1 Research Institute for Primary Care and Health Sciences, Keele University, ST5 5BG Keele, Staffordshire UK; 2 School of Psychology, Keele University, Keele, Staffordshire UK; 3 University Hospital of North Midlands, Stoke on Trent, Staffordshire UK; 4 Keele University, Keele, Staffordshire UK; 5 Psychological Sciences, University of Manchester, Manchester, UK

**Keywords:** Feasibility study, Crossover, Medication withdrawal, Pramipexole PR, Ropinirole MR, Idiopathic Parkinson’s disease, Cued recall, Safety processes, Acceptability, Barriers to participation

## Abstract

**Background:**

The aim was to assess the feasibility of a single-centre, single-blind, randomized, crossover design to explore the effects of two slow-release dopamine agonists, ropinirole and pramipexole, on cued recall in Parkinson’s disease.

As the design required a switch from the prescribed agonist (pramipexole-to-ropinirole, or ropinirole-to-pramipexole), the primary objectives were to (a) examine the efficacy of processes and procedures used to manage symptoms during the washout period and (b) to use cued recall estimates to inform a power calculation for a definitive trial.

Secondary objectives were to assess consent and missing data rates, acceptability of clinical support for the OFF sessions, experience of the OFF sessions and of agonist switching, barriers-to-participation for patients and informal caregivers.

**Methods:**

Patients were randomized in a 1:1 ratio to two treatment arms and stabilized on each agonist for 6 weeks. The arms differed only in the sequence in which the agonists were administered. Cued recall was assessed ON medication and, following a washout period resulting in 93.75% agonist elimination, OFF medication.

**Results:**

A total of 220 patients were screened: 145 were excluded and 75 invitations to participate were sent to eligible patients. Fifty-three patients declined, 22 consented and 16 completed the study.

There were no serious adverse events, and rates of non-serious adverse events were equivalent between the agonists.

Using the largest standard deviation (SD) of the ON–OFF difference cued recall score (inflated by ~25% to give a conservative estimate of the SD in a definitive trial) and assuming an effect of at least 10% of the observed range of OFF medication cued recall scores for either agonist to be clinically important, a main trial requires a sample size of just under 150 patients.

The consent and missing data rates were 29 and 27% respectively. The washout period and the preparation for the OFF sessions were acceptable, and the sessions were manageable. The experience of switching was also manageable. Barriers to participation included concerns about disease stability, side effects, research process, carer workload and accessibility of the information sheet.

**Conclusions:**

This study presented challenges to recruitment both in design and execution, and while it was a major aim of the study to assess this, evaluation of these challenges provided the opportunity to explore how they could be overcome for future studies.

**Trial registration:**

EudraCT 2012-000801-64

## Background

Around 10,000 new cases of Parkinson’s disease (PD) are diagnosed each year, which indicates that around 1% of the UK population (an estimated 122,795 people) over the age of 60 years have PD; this figure is set to rise by an estimated 28% by 2020 as the population ages [1–4]. PD is diagnosed according to the presence of motor features (tremor, rigidity, slowed movement) caused by the loss of dopamine-producing cells in the midbrain [5–9].

Clinical management is focused on using dopaminergic replacement drugs to alleviate the cardinal motor signs. These drugs remediate motor features by increasing availability of dopamine using levodopa and/or monoamine oxidase inhibitors, and/or by mimicking the action of dopamine using second-generation dopamine agonists such as immediate- or slow-release preparations of pramipexole or ropinirole [10–15].

Dopaminergic medication can also have a significant effect on cognitive function. Evidence indicates that the requisite dopaminergic state necessary to control motor signs has the potential to move a patient away from his or her optimum for certain cognitive functions [16–20]. The relationship between the efficiency of neuronal activity and the state of dopaminergic modulation in the l-dopa overdose hypothesis is represented by a Yerkes–Dodson inverted U-shaped curve, with cognitive functions declining with deviation away from optimum dopamine levels, indicated by the centre of the curve. Extrapolating this model to cued recall implies that dopaminergic medication has the capacity to both improve and impair this kind of memory, depending on baseline dopamine levels in the underlying neural circuitry.

We have recently reported a series of studies in which cued recall of recently experienced events is impaired in medicated patients with PD [21–25]. Interpreting these findings in the context of the l-dopa overdose hypothesis theory implies at least two competing explanations: either dopaminergic medication is overdosing ‘relatively normal’ dopamine levels in those brain areas supporting memory or dopaminergic drugs are reducing the severity of a PD-related dopamine depletion in the brain areas supporting memory.

We explored these alternative predictions in an open-label, nonrandomized pilot of 24 patients with PD in the early (*n* = 12) and moderate (*n* = 12) disease stages [26]. The patients’ memory was compared to that of a group of 24 matched healthy controls. To compare the effects of medication on memory, testing took place when the patients were in a medicated or ‘ON’ state, and also on a different occasion following a 24-hour washout period before the patients took their next medication, and so were in an unmedicated or ‘OFF’ state. Memory was also assessed on 2 occasions in the healthy controls, who were not taking any medication, to control for practice effects. The pilot revealed three key findings:

Firstly, compared to the healthy control performance, the PD group exhibited a cued recall deficit when tested OFF medication, indicating a PD-related memory decline.

Secondly, the severity of the patients’ memory decline increased when the same patients were tested ON medication, suggesting that dopaminergic medication increases the severity of a PD-related decline in memory.

Thirdly, the ON medication decline in memory was most pronounced in a subset of patients medicated with the second-generation non-ergot dopamine agonist pramipexole prolonged-release (abbreviated subsequently to pramipexole), with patients on the second-generation non-ergot dopamine agonist ropinirole modified-release (abbreviated to ropinirole) showing a less severe decline. These preliminary findings were replicated in a further open-label nonrandomized, parallel-group study of 21 patients with PD medicated with either pramipexole or ropinirole [27].

While these were intriguing findings that had not previously been reported in either the scientific or medical literature, the study design meant that the effects of confounding variables, such as demographic and or clinical differences between the patients on either pramipexole or ropinirole, could not be ruled out.

Thus, the aim of this study was to assess the feasibility of a crossover design to explore the effects of pramipexole and ropinirole on memory in patients with idiopathic PD without cognitive impairment.

As the design required patients to switch from their prescribed dopamine agonist (pramipexole-to-ropinirole, or ropinirole-to-pramipexole), the primary objectives were to (a) examine the efficacy of processes and procedures used to manage symptoms during the washout period and (b) to use cued recall estimates to inform a power calculation for a definitive trial.

The secondary objectives were to assess (i) rates of consent and missing data; (ii) acceptability of the support provided for the OFF medication sessions and whether the OFF session itself was manageable; (iii) the experience of switching between the investigational medicinal products (IMPs), i.e. ropinirole and pramipexole, assessed at the mid- and end-of-study clinic visits and in the semi-structured interview administered at the end of the trial; and (iv) barriers to participation for eligible patients and informal carer givers.

## Methods

The reporting of the study follows the CONSORT statement recommendations [28].

### Design

This 24-month feasibility study was a single-centre, single-blind, randomized study of two IMPs, ropinirole and pramipexole, in male and female patients with a confirmed primary diagnosis of idiopathic PD without cognitive impairment.

Patients were recruited on one of these agonists, and randomized in a 1:1 ratio to two treatment arms. The arms differed only in the sequence the IMPs were administered (pramipexole then ropinirole, or ropinirole then pramipexole).

Patient-participants commenced their first trial IMP without a ‘run-in’ or washout period. They were stabilized on this IMP for 6 weeks, and then, their memory was tested on two separate occasions in the following 2 weeks: in one session, the memory was tested 90 min after taking the IMP (ON), and on a separate occasion, the memory was assessed following 24–48 h of IMP withdrawal (OFF).

The OFF session was preceded by a withdrawal period that resulted in 93.75% (four half-lives) IMP elimination. After four half-lives, the amount of drug remaining (6.25%) is considered to be negligible regarding its therapeutic effects [29].

The order of ON and OFF sessions was counterbalanced.

**Fig. 1:**
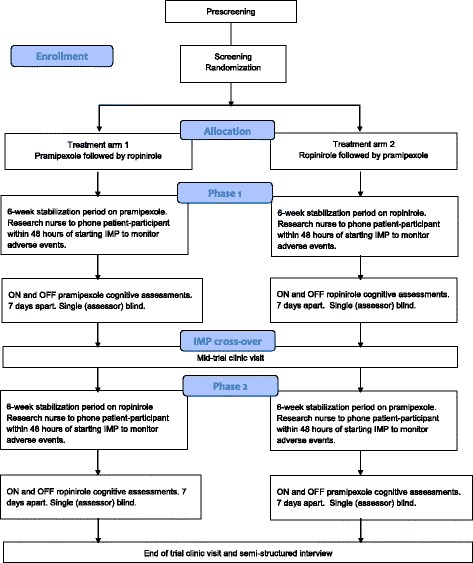
CONSORT diagram showing key stages of study design

A CONSORT diagram showing key stages of the study design is presented in Fig. 1.

### Clinical trial authorization

Clinical trial authorization under The Medicines for Human Use (Clinical Trials) Regulations 2004 S.I. 2004/1031 was received from the Licensing Authority in November 2012.

### Ethical approval and consent

The study was conducted in accordance with the ethical principles that have their origins in the Declaration of Helsinki. The patients were sent approved participant invitation letters that included a reply slip and an information sheet for consideration. If a patient indicated interest by sending a positive reply slip, he/she was contacted by the research nurse and invited to a screening session with the chief investigator, who was also the principal investigator in this study.

Written informed consent was obtained at the screening interview by the chief investigator, with the research nurse present as the patient advocate. Patients were given an opportunity to ask any questions. Consent was obtained before any study procedures took place.

### Recruitment

Recruitment commenced in April 2013. The target population was males and females aged 50–80 years with idiopathic, sporadic PD as determined by the UK Parkinson’s Disease Society Brain Bank Clinical Diagnostic Criteria, with a Hoehn–Yahr disease severity stage of 1–4 [30].

The target number of complete datasets was 50. The sample size was determined in terms of obtaining sufficiently precise estimates (as represented by the width of a 95% confidence interval) of key parameters that would inform a power calculation for a subsequent main trial. A figure of 50 is supported by reports on sample sizes for pilots with continuous outcomes [31, 32].

Eligible patients were identified from the sponsor’s (University Hospital of North Midlands NHS Trust) Department of Neurology using the PD database and clinic lists, the GPs in Staffordshire (acting as participant identification sites), the chief investigator’s private practice, local pharmacies, advertisements in local newspapers and recruitment talks given to local PD associations.

Patients entered the study on one of the two IMPs within the approved dosage range. The maximum dosage of each IMP, as specified in the summary of product characteristics, was pramipexole 0.52–3.15 mg once daily and ropinirole 2–12 mg once daily [33, 34].

Patients were maintained on the same or equivalent therapeutic dose for the stabilization periods and for the ON medication research session, using published conversion norms, unless clinical indications suggested modification [35].

### Randomization procedure

Once the consent form had been signed, the chief investigator randomized the patient to the treatment arms.

Third-party randomization, in a 1:1 ratio, took place during the screening clinic visit using a locked system that automated the random assignment of treatment groups to randomization numbers. The third-party randomization protocol was a web-based programme, hosted by Keele University, and was on a password-protected site.

The patient’s randomization code was recorded in the medical records and case report form. This information was stored on-line, was read-only and could be accessed by the chief investigator and research nurse at any point during the trial.

### Level and method of blinding

During the trial, the patients and the clinical members of the research team (chief investigator, research nurse) were not blind to the medication codes. The patients could not be blinded as they saw the physical nature of the tablets, and the chief investigator and research nurse managing the patients needed to know how to advise the patients at the beginning of each treatment phase of the trial, and when switching between the IMPs, and how to prepare for the medication withdrawal period.

Only the research assistant administering the cued recall memory test during the ON and OFF research sessions was blind to the medication codes. The medication codes were not made available to the research assistant until each patient had completed the trial, with his/her data scored and entered into the database.

### Outcomes

The two primary outcomes were efficacy of processes and procedures used to manage symptoms during the washout period (measured in relation to adverse events) and estimates of cued recall performance (to inform the sample size calculation). The secondary outcomes were rates of consent and missing data, acceptability of the support provided for the OFF medication sessions and whether the OFF session itself was manageable, the experience of switching between IMPs (assessed at the semi-structured interview administered at the end of the trial) and barriers to participation for eligible patients and informal carer givers (semi-structured interview).

### Enrolment and study visits

Patients completing the feasibility trial were intended to participate for a maximum period of no longer than 16 weeks. During this period, they were involved in eight separate sessions:

- Three involved the chief investigator and research nurse and took place during research-dedicated clinics in a UHNM research facility:
  - ○ Screening visit in week 1 (visit 1)
  - ○ Mid-study follow-up appointment, taking place between weeks 6 and 8 (visit 4)
  - ○ An end-of-study follow-up appointment, between weeks 14 and 16 (visit 7)
- Four involved the research assistant and took place in the patient’s home, around 9 or 10 a.m. in the morning:
  - ○ Separate ON and OFF medication sessions for the first IMP, between weeks 6 and 7 (visits 2 and 3)
  - ○ Separate ON and OFF medication sessions for the second IMP, between weeks 13 and 15 (visits 5 and 6)
- One visit with the research assistant followed the end-of-study clinic visit, for the purposes of completing a semi-structured interview about participation, between weeks 14 and 16 (visit 8).

For the ON research session, medication was taken as usual roughly 60 min before the session commenced. For the OFF session, medication was delayed prior to the session. Medication was resumed following completion of the session, according to criteria discussed with the chief investigator. The ON medication and OFF medication sessions for each drug should have taken place during a 7-day window, on separate days separated by at least 3 days. The order of the ON and OFF research sessions was determined by the randomization programme.

### Washout periods

A single washout period, between the first and second IMP, was used to eliminate a carryover effect. The elimination half-life for each drug is 8–12 h for pramipexole in older patients, falling at the upper end of the range: 6 h for ropinirole and 3 h for l-dopa and monoamine oxidase B inhibitors.

To achieve 93.75% elimination of each drug (equating to four half-lives), the following washout periods were required: pramipexole, 48 h (4 × 12 h); ropinirole, 24 h (4 × 6 h); l-dopa and monoamine oxidase B inhibitors, 12 h (4 × 3 h). At the mid-study clinic visit, patients were given the second IMP, which they took until the end-of-study visit.

During the washout period, patients continued to take l-dopa and/or monoamine oxidase B inhibitor as usual. The half-lives of these drugs are shorter than those of the IMPs, and therefore, the washout period required for 93.75% elimination of l-dopa and monoamine oxidase inhibitor (half-life is between 2 and 3 h) is 12 h. l-dopa and monoamine oxidase inhibitors were withdrawn 12 h (overnight) before the OFF research session. The OFF session took place in the morning, following a washout period resulting in four half-lives of the drug being eliminated (i.e. 93.75%).

### Data security and monitoring

A research risk assessment of the feasibility trial was carried out by the chief investigator and the regulatory research associate from the sponsor’s research and development department, prior to the preparation of the protocol. The first risk assessment was assessed as moderate by 1 point initially (1 point over the low to moderate risk boundary), but reassessed later as low-risk, requiring minimal mitigation.

Validation procedures included regular data entry checks performed on a minimum of 25% of complete patient data by the monitor. All source data and trial documentation were available for trial-related monitoring. The patients consented for this within the consent process.

Patients’ contact details were disclosed to the research assistant, for the purposes of arranging the four home visits to complete the memory assessment. All patient data collected by the research assistant were identified using a unique study code. This same code was used to enter patient data into the Microsoft Excel^©^ database. Copies of signed informed consent forms were placed in the patient’s medical records and originals retained in the trial file and kept separate from the data.

Monitoring procedures were carried out by the sponsor and were in accordance with standard operating procedures. The monitor checked the case report forms, trial master file and other trial documentation for completeness and the compliance of the trial team with the protocol and good clinical practice (GCP) standards. The initial monitoring visit occurred before the trial started to ensure that the trial complied with the protocol and GCP standards. Further monitoring visits took place at regular intervals (approximately twice per year), with a final monitoring visit conducted to close the trial.

### Study committees

A steering group was formed comprising a lay chair, lead researcher (Edelstyn), chief investigator (Ellis), research nurse representatives, research assistant (Shepherd), pharmacy representatives, research and development department representatives (Longshaw, Watts) and up to four patient-public representatives.

Membership of the operational group included the lead researcher (chair), chief investigator, research nurse representatives, research assistant, trial pharmacists, sponsor research and development representatives. The operational group met on a monthly basis.

## Analytic strategy

### Quantitative data

As this was a feasibility study, no hypothesis tests were performed. Instead, 95% confidence intervals (CIs) are presented to indicate the precision of calculated estimates and to present a range of plausible alternative values for these estimates.

### Qualitative data

All patients who completed the trial were invited to participate in semi-structured interview to reflect on their experience of participation.

A barriers-to-participation study was introduced 9 months into the 18-month recruitment period to explore reasons for poor recruitment. NRES permission for retrospective contact of decliners prior to receiving approval was not obtained. There was no a priori selection of decliners for interview. All those who agreed were interviewed.

Interview recordings were transcribed by the research assistant. During the transcription process, initial thoughts and observation were noted. Accuracy was checked by listening to each recording while reading through the respective transcript, and any necessary amendments were made. The interviews were transcribed into Microsoft Word^©^ and were analysed using thematic analysis [36].

Initial topic coding was achieved by reading through the transcripts and detailing basic themes within the text using the Microsoft Word^©^ comment function. New themes emerged from the data throughout this process, and the procedure was therefore repeated to ensure that text from transcripts that had been read prior to newly developed themes could be subsumed under the appropriate theme.

## Results

### Recruitment and retention

A total of 220 patients with PD were screened for eligibility (number of patients with PD found among neurology patients screened). One hundred and forty-five were excluded, and 75 eligible patients were invited to take part. Fifty-three patients declined, 22 consented and 16 completed the study. The age distribution at the time of consent is shown in Table 1.

**Table 1:** Age distribution (at time of consent)

Age (years)	Frequency
51–55	2 (female = 1, male = 1)
56–60	4 (female = 1, male = 3)
61–65	6 (female = 2, male = 4)
66–70	3 (female = 1, male = 2)
71–75	5 (female = 1, male = 4)
76–80	2 (male = 2)

**Fig. 2:**
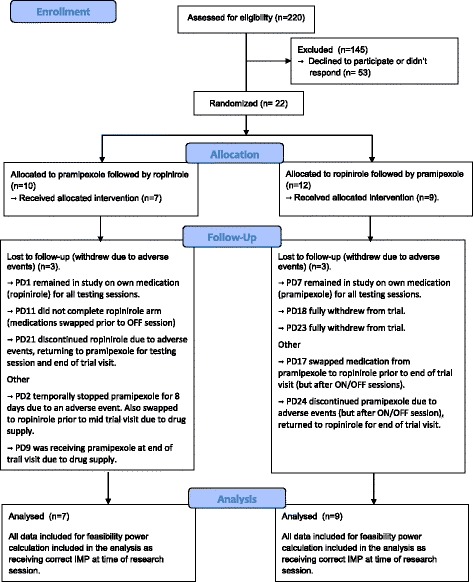
CONSORT diagram showing the number of patients assessed for eligibility, randomized, follow-up and complete datasets analysed

Information on the number of patients assessed for eligibility, randomized and discontinued (together with reasons) and the complete datasets analysed are presented in a CONSORT diagram in Fig. 2. Ten patients were randomized to treatment arm 1 (pramipexole then ropinirole) and 12 to treatment arm 2 (ropinirole then pramipexole).

### Outcomes

No serious adverse events were reported. Effects of short-term withdrawal of the IMPs were minimal when assessed at the mid- and end-of-study visits. A total of 84 non-serious adverse events were reported (plus one report of medication error), with seven participants experiencing adverse events; as a result, two withdrew from the study and three were swapped back to their original dopamine agonist but agreed to stay in the study. There was one additional case of the IMP being temporarily stopped due to an adverse event. This enabled the testing and refinement of adverse event handling and reporting by the clinical members of the research team. In terms of adverse events (non-serious events considered to be related to the IMP by the chief investigator), the rate was very similar between both groups (pramipexole, *n* = 28; ropinirole, *n* = 30).

Allocation to treatment arm, IMP daily dose, duration of IMP treatment and details of compliance for each patient are provided in Table 2. Arm 1 had 3 patients who discontinued their assigned treatment due to non-serious adverse events (PD1, PD11, PD21). These patients were switched to their original dopamine agonist but agreed to remain in the study to continue the study processes. Other patients who deviated from their assigned IMP were PD2 (pramipexole was temporarily stopped for 8 days, and the patient switched back to own pre-study supply of ropinirole prior to the mid-study visit with the chief investigator) and PD9, who ran out of study medication prior to the mid- and end-of-study visits and took own supply (however, this patient was receiving the correct IMPs for the testing sessions). Therefore, PD2, PD6, PD9, PD10, PD14, PD16 and PD19 fully completed the study.

**Table 2:** Pre-study dopamine agonist, allocated treatment arm, first and second IMP and daily dose and duration of IMP treatments

Patient study code	Pre-study dopamine agonist	Trial arm	First IMP	Daily dose (mg)	Number of days on IMP	Second IMP	Daily dose (mg)	Number of days on IMP
PD1	RPR	1	PPX	2.1	3	RPR^a, b^	8	134
PD2	RPR	1	PPX	2.62	58	RPR	10	48
PD3	RPR	2	RPR	6	51	PPX	1.57	58
PD4	RPR	2	RPR	4	40	PPX	1.05	55
PD5	Consented but then withdrew before taking first IMP							
PD6	PPX	1	PPX	2.1	64	RPR	8	52
PD7	PPX	2	RPR	10	13	PPX^a, b^	2.62	97
PD8	RPR	2	RPR	12	54	PPX	3.15	62
PD9	PPX	1	PPX	1.57	62	RPR	6	56
PD10	RPR	1	PPX	2.1	53	RPR	8	68
PD11	PPX	1	PPX	1.05	48	RPR^b^	4	30
PD12	PPX	2	RPR	10	52	PPX	2.62	56
PD13	Consented but then withdrew before taking first IMP							
PD14	PPX	1	PPX	0.52	52	RPR	2	37
PD15	Consented but then withdrew before taking first IMP							
PD16	PPX	1	PPX	1.57	63	RPR	6	55
PD17	PPX	2	RPR	6	60	PPX	1.57	37
PD18	PPX	2	RPR	6	3	Withdrew from IMP and study		
PD19	RPR	1	PPX	2.1	55	RPR	8	84
PD20	PPX	2	RPR	8	66	PPX	2.1	41
PD21	PPX	1	PPX	3.15	100	RPR^b^	12	7
PD22	RPR	2	RPR	6	55	PPX	1.57	48
PD23	PPX	2	RPR	8	5	Withdrew from IMP and study		
PD24	RPR	2	RPR	6	58	PPX	1.57	37
PD25	RPR	2	RPR	2	68	PPX	0.52	62

*PPX* pramipexole PR, *RPR* ropinirole

^a^Withdrew from study and reverted to pre-study dopamine agonist

^b^Discontinued their assigned treatment due to non-serious adverse events, reverted back to pre-study agonist but agreed to remain in study to test study processes

Arm 2 had 3 patients who discontinued their assigned treatment due to non-serious adverse events (PD7, PD18 and PD23). Participant PD7 reverted to his original dopamine agonist due to adverse events but agreed to remain in the study to continue the study processes. However, PD18 and PD23 wished to fully withdraw and therefore had no further study assessments or visits. Other participants who deviated from their assigned IMP were PD24, who discontinued pramipexole due to non-serious adverse events but after the ON/OFF research session (therefore, this participant was on ropinirole, not pramipexole, for the end-of-study visit), and PD17, who switched medications from pramipexole to ropinirole prior to the end-of-study visit (but after the ON/OFF session due to attending a private (non-research) appointment). Therefore PD3, PD4, PD8, PD12, PD17, PD20, PD22, PD24 and PD25 fully completed the study.

In total, therefore, 16 participants completed the study (defined as completing the memory assessment sessions on their assigned IMP) and 5 completed the end-of-study interviews with the research assistant.

**Fig. 3:**
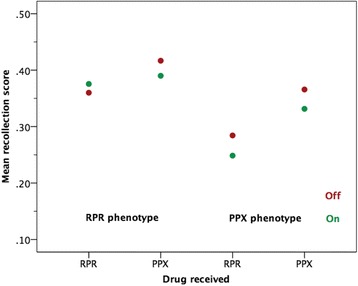
Effect of pramipexole (PPX) versus ropinirole (RPR) on cued recall according to patients’ dopamine agonist on entry to the study

Owing to the small sample size, the estimates from the study are necessarily imprecise (as reflected in wide confidence intervals). However, the data suggest that the effect of pramipexole on cued recall (OFF–ON difference score) is 0.030 (95% CI −0.192, 0.079). The corresponding effect of ropinirole is 0.007 (95% CI −0.070, 0.084), indicating a somewhat smaller decrement in recall. It also appears, from Table 3 and Fig. 3, that patients entering the study on pramipexole are susceptible to both a pramipexole-related (OFF–ON difference score 0.034, 95% CI −0.067, 0.135) and ropinirole-related (OFF–ON difference score 0.036, 95% CI −0.144, 0.215) decrement in cued recall. For those entering the study on ropinirole, pramipexole (OFF–ON difference score 0.027, 95% CI −0.038, 0.091) showed a decrement in cued recall, but this did not occur with ropinirole (OFF–ON difference score −0.016, 95% CI −0.092, 0.061).

**Table 3:** Effect of pramipexole versus ropinirole on cued recall according to patients’ pre-study dopamine agonist

Pre-study dopamine agonist	Drug	Condition	Estimate: mean (SD)	Difference (OFF–ON)	
				Mean (SD)	95% CI
PPX (*n* = 7)	PPX	OFF	0.366 (0.137)	0.034 (0.109)	−0.067, 0.135
		ON	0.331 (0.140)		
	RPR	OFF	0.284 (0.152)	0.036 (0.194)	−0.144, 0.215
		ON	0.249 (0.173)		
RPR (*n* = 9)	PPX	OFF	0.417 (0.162)	0.027 (0.084)	−0.038, 0.091
		ON	0.390 (0.168)		
	RPR	OFF	0.360 (0.155)	−0.016 (0.100)	−0.092, 0.061
		ON	0.376 (0.147)		

*PPX* pramipexole PR, *RPR* ropinirole, *SD* standard deviation, *CI* confidence interval

If the effects of pramipexole and ropinirole are compared (pramipexole minus ropinirole), there is a small difference favouring ropinirole (0.023, 95% CI −0.088, 0.134). A crossover trial is potentially subject to a period effect, and this was assessed by comparing the difference in effect of the two IMPs, between the first and second period. The difference was 0.022 (95% CI −0.094, 0.138). The estimate of pramipexole versus that of ropinirole adjusted for the period effect is slightly smaller than the crude estimate, 0.020, 95% CI −0.095, 0.136.

Accordingly, in addition to an analysis adjusting for a period effect, it would be advisable to utilize blocked randomization in a future trial, whereby equal numbers of patients would be allocated to each treatment sequence, helping to offset any period effect. This also raises the issue of including an additional washout period, prior to administration of the first treatment, in order to remove the potential effect of pre-randomization IMP.

The standard deviation (SD) of the within-subject differences between the two IMPs was 0.208 (95% CI 0.154, 0.322). If this is inflated by approximately 25% to give 0.260 as a conservative estimate of the SD in a subsequent definitive trial [30], Table 4 gives the numbers of subjects required to detect a range of differences in a fully powered RCT, with statistical power of 0.90 and a two-tailed significance level of *p* ≤ 0.05.

**Table 4:** Sample sizes for a main study according to a range of effects

Sample size	Mean difference	Standardized effect: mean difference/(SD/√2)
25	0.176	0.958
50	0.122	0.662
75	0.099	0.536
100	0.085	0.463
125	0.076	0.413
150	0.069	0.377
175	0.064	0.348
200	0.060	0.326
225	0.056	0.307
250	0.054	0.291
275	0.051	0.277
300	0.049	0.266

*SD* standard deviation

The minimum and maximum OFF medication cued recall scores for either ropinirole or pramipexole were 0.040 and 0.735, giving a range of 0.695. Assuming an effect of at least 10% of the observed range of scores (0.070) to be clinically important, a main study would therefore require a sample size of just under 150, at the above levels of power and statistical significance (without accounting for loss to follow-up).

### Secondary outcomes

With regard to the secondary objectives, the consent and missing data rates were 29% (95% CI 19%, 40%) and 27% (95% CI 9%, 46%), respectively.

The experience of participation from the end of study interviews with 5 patients completing the study revealed two main themes, centred on the experience of participation and the motivation to take part. The washout period itself, as well as the preparation for the OFF sessions, were acceptable, and the experience of switching between the IMPs was manageable. Two main themes were identified: the experience of participating and motivation to take part. Both themes, together with each of the subthemes, are presented in Table 5. All patients contributed to each of the two main themes, but not necessarily to all subthemes.

**Table 5:** The main themes and subthemes and illustrative quotations from the end-of-study interviews with 5 patients

Experience of participating	*Patient information sheet was too long and complicated.* ‘Yes, I thought so, I let my husband read it too, but yes, I thought so […] I think there was a lot of technical language in it, maybe making that clearer would help? […] I guess some people might get lost in it maybe?’ (patient-participant 2). *The preparation for the OFF medication sessions was acceptable, and the sessions themselves were manageable.* ‘The OFF days were interesting to see what I would be like but, not too concerning. It’s basically like when you forget to take a tablet or miss one by accident you know, which happens I can tell you, and then I will be out somewhere and will realize […] it was fine, really, it’s no different to when I forget to take them. You slow down a bit, but you take more care don’t you? […] yes [*it was manageable*].’ (patient-participant) ‘To be honest, I could tell I was slower, but other than that there isn’t that much difference really. I was careful, when walking around the house because I knew I was slower.’ (patient-participant 8). *The experience of switching was acceptable to participants.* ‘It was okay. I think when I switched there was a day where I was a bit worse than normal, but that could have been when I was coming off one and starting the next I suppose couldn’t it? But then the following day I was back to my normal self actually. I didn’t really have any problems.’ (patient-participant 6) *Participation did not have a major impact on the caregiver.* ‘Oh not at all. I mean we talked about it before I decided to take part, obviously, as you would. But it didn’t do anything to him really. I mean, I like to be as independent as I can, while I can.’ (patient-participant 2) ‘No not really, she came with me to the hospital for those times. But I think that is it. She reminded me of the appointments as well […] Yeah, it’s because she is in control of the diary you see?’ (patient-participant 3)
Motivation to take part	*Helping someone else.* ‘So we need to do these, going back, we are benefitting from things that others have done for us and we can pass that forward now, for future people. […] I thought it would be good to contribute something back you know.’ (patient-participant 2) ‘To help someone further down the line […] Because that is why we’ve got the medications we’ve got now, because those have done it before.’ (patient-participant 12) *Trying a new drug may help their symptoms.* ‘No, not really. I was interested to see if the other drug would be better for me, whether I would see any improvement, or whether I would be worse. Because, I had never tried it before. I was interested to know.’ (patient-participant 6) ‘I hadn’t really thought about it like that, I was hoping it would improve my walking and make me a bit better actually, as it happened, I couldn’t really tell the difference at all.’ (patient-participant 8) *Concern about their own memory and recognition of the importance of memory research.* ‘That’s why I took part, and have taken part previously. Memory is so important, especially to me […] it feels awful when I forget little things. It’s just little things. But it gets to me […] Well people’s names, is frustrating, you know it too, it might be someone you know really well. You know what I mean?’ (patient-participant 6) ‘I think about my memory a lot. It’s why I took part […] You want to know if it is getting worse don’t you? Especially if it’s worse on one tablet […] People don’t really think about that, do they? I would want know if they were making my memory worse.’ (patient-participant 8)

Finally, barriers to participation were explored with 5 patients who declined to take part in the main study and 5 informal carer givers. Four main themes emerged, which focused on medication concerns relating to stability and side effects, accessibility of the recruitment materials, fear of the unknown and carer workload. The four main themes together with each of the subthemes, along with illustrating quotations, are presented in Table 6. All participants contributed to each of the four main themes, but not necessarily to all subthemes.

**Table 6:** The main themes and subthemes and illustrative quotations from the barriers-to-participation interviews with 5 patients who declined to take part and 5 informal caregivers

Medication concerns	*A risking to stability:* ‘It takes a long time to get stable, I didn’t want to mess with that’ (patient 3). *Previous switching experience*: ‘I felt terrible for 6 months’ (patient 1); ‘It was quite hard, I was quite poorly’ (patient-participant 3); ‘I didn’t want to start having side effects again’ (patient 4). *Irreparable deterioration*: ‘these [tablets] allow us to live our lives. You don’t to start messing with that in case you can’t get it back (patient 4).
Accessibility of materials	*Patient information sheet*: ‘It was a bit ‘sciencey” (informal caregiver 1); ‘it was long […] it took some effort to read’ (informal caregiver 2); ‘it is too complicated’ (patient-participant Inconvenience: ‘the hospital trips, we weren’t keen on the driving (px1); with all the visits, fitting it all in would have been difficult for us (informal caregiver 2).
Fear of unknown	*Research process*: ‘I thought that it was quite a complex study’ (patient-participant 2); ‘I thought it meant testing brand new drugs’ (patient 4). *Research team*: ‘I think there is a trust thing isn’t there’ (informal caregiver 3); ‘I suppose others would be more comfortable’ (patient 3).
Caregiver workload	‘Her getting worse was a problem for me, because it would be a problem for me’ (informal caregiver 3). ‘If I got worse then he would have to help get about you see. I don’t like to ask him to do more for me’ (patient 3).

## Discussion

This study presented challenges to recruitment in both design and execution, and while it was a major aim of the study to assess this, evaluation of these challenges also provided the opportunity to explore how these could be overcome for future studies in this area. The study necessitated the use of different methods and approaches to identify and recruit eligible patient-participants. This included raising awareness using promotional material in local newspapers and pharmacies and establishing links with GP surgeries (Participant Identification Centres) through the Primary Care Research Network. However, the recruitment target of 50 was not met. Consequently, estimates of key parameters are somewhat imprecise; in particular, the wide confidence interval around the estimated treatment effect between the two IMPs suggests that this effect could plausibly lie between approximately –0.10 and 0.14; the suggested minimum clinically important effect of 0.70 lies within this range but is somewhat larger than the point estimate of approximately 0.20. The small sample size also means that the probability of detecting adverse events is reduced [37].

The challenges and mitigations implemented included the development of a suite of study-specific standard operating procedures, including a named standard operating procedure controller. Infrastructure to support clinical trials of medicinal products was not optimum at study start-up. The organization underwent significant changes prior to and during the study. We now have clinical studies officers in place to support study set-up, which includes early engagement with support departments and academics that lead clinical trials of medicinal products (this includes a full risk assessment by the sponsor, which is now used to support the decision regarding capability/capacity to sponsor clinical trials of medicinal products). There are also dedicated study monitor roles within the department. The barriers-to-participation study identified four main themes: concerns that changing medication may worsen their PD symptoms and also fears about side effects; trial accessibility, e.g. the information sheet was long and too technical; fear of the research process; and worries about increasing the work load of spouses/informal carers.

Recruitment challenges are particularly relevant in older populations [38], and the heterogeneous nature of PD means that achieving a sufficient homogeneous patient sample requires the implementation of very effective recruitment strategies. Individuals with PD are typically elderly and disabled; therefore, participation in clinical trials often requires the assistance of a caregiver, not only with hospital visits and organizing medication but also in the decision to participate itself.

For drug trials in particular, patients with PD have positive perceptions of placebo-controlled trials, although they are strongly in favour of receiving the active agent [39]. Furthermore, evidence suggests a preference for trial designs that include two active agents (or an active comparator) over placebo-controlled designs and indicates that this may facilitate recruitment due to patients being less likely to experience ‘lessebo’ effects (the negative expectations associated to the administration of a placebo) [40].

The challenges of conducting research in PD are illustrated by a recent on-line 5-item survey by Mathur et al. [41]. Their aim was to explore patients’ barriers to participation and researchers’ challenges in conducting drug trials in PD. From an estimated sample size exceeding 10,000 people, the proportion of respondents was around 3%. A total of 33 responses were from researchers and 263 from patients. From the researcher perspective, the main concerns were related to insufficient financial and administrative support. Of greater interest to our study was the overlap with Mathur et al.’s patient-reported barriers: fear of potential adverse consequences, misconceptions regarding clinical trial systems, and issues related to a perceived lack of communication of relevant information between the research and patient communities.

To address the generic as well as specific recruitment problems discussed above, we recommend adoption of the the ‘QuinteT Recruitment Intervention’ (QRI), which aims to understand recruitment as it happens and then develop an action plan to address identified difficulties and optimize informed consent in collaboration with the CTU and chief investigator [41]. The QRI uses a combination of standard and innovative qualitative research methods with some simple quantification to understand recruitment and identify sources of difficulties.

## Conclusion

Recruitment rates to the main feasibility study were disappointing. Patient and informal caregiver concerns clearly influenced this and can, at least to some extent, be mitigated by improved communication, reassurance, exposure to the research team and awareness-raising. The sponsor now has improved capability to support CTIMPs. Going forward, the sponsor together with the research team is in a position to mitigate these challenges in a subsequent main trial, particularly in collaboration with a Clinical Trials Unit. Implementing these measures, together with integration of the QRI from the outset in order to prevent difficulties developing, and optimizing recruitment from the start, rather than applying it to an ongoing RCT experiencing recruitment shortfalls where time to rectify/address recruitment difficulties may be limited, and increase confidence that realistic recruitment targets, while still challenging, could be achieved.

Delivering safe, effective and cost-effective pharmacological management of PD is a priority. It is important to communicate effectively to the patient community the importance of drug trials in developing new treatments and to engage patients and informal caregivers in the communication and promotion of research.

It is important to note that this feasibility study does not provide and did not seek to provide firm evidence that dopaminergic medication affects memory. Therefore, people with PD should not change their medication based on the results of this study.

## Abbreviations

CI: Confidence interval
GCP: Good clinical practice
IMP: Investigational medicinal product
PD: Parkinson’s disease
SD: Standard deviation
